# COVID-19 mRNA vaccines: a prospective outlook from technological innovation to clinical practice

**DOI:** 10.3389/fimmu.2026.1757291

**Published:** 2026-06-04

**Authors:** Junqi Ma, Huoying Chen, Yunyi He, Yuye Huang, Xianlong Duan, Qiliang Liu, Zheng Liu, Hongbo Liu

**Affiliations:** 1 Department of Laboratory Medicine, The Second Affiliated Hospital of Guilin Medical University, Guilin, China; 2 Genetics and Precision Medicine Laboratory, The First Affiliated Hospital of Guilin Medical University, Guilin, Guangxi, China; 3 Guangxi Key Laboratory of Multimodal Biomarkers and Precision Diagnosis, Guilin Medical University, Guilin, Guangxi, China; 4 School of Laboratory Medicine and Biotechnology, Guilin Medical University, Guilin, Guangxi, China

**Keywords:** COVID-19, mRNA vaccines, vaccine effectiveness, immune response, lipid nanoparticles

## Abstract

The COVID-19 pandemic established mRNA vaccines as a clinically validated platform for rapid vaccine development and deployment. This review summarizes recent progress in COVID-19 mRNA vaccine technology, clinical performance, immunological mechanisms, and translational applications. First-generation nucleoside-modified mRNA vaccines formulated in lipid nanoparticles demonstrated strong protection against symptomatic disease and, more durably, against severe outcomes, while variant-driven immune escape, waning protection against infection, limited mucosal immunity, and heterogeneous responses in special populations revealed important constraints. The review compares mRNA vaccines with other COVID-19 vaccine platforms and clarifies endpoint-specific correlates of protection, emphasizing the distinct roles of neutralizing antibodies, memory B cells, T-cell responses, and non-neutralizing antibody functions. It further examines unresolved issues associated with repeated vaccination, including immune imprinting and IgG4 class switching, and evaluates technological strategies designed to improve durability, breadth, delivery, and immune programming. Key innovations include optimized RNA chemistry, structure-guided antigen design, advanced lipid nanoparticle formulations, alternative delivery systems, immune-shaping adjuvant approaches, and next-generation RNA formats such as self-amplifying RNA and circular RNA. Finally, the review discusses vaccination strategies for immunocompromised individuals, pregnant and lactating women, older adults, and children, as well as the expansion of mRNA technology into respiratory virus vaccines, cancer immunotherapy, and therapeutic protein expression. These developments define mRNA technology as a modular platform whose clinical impact depends on aligning RNA architecture, delivery system, antigen design, and target population.

## 1 Introduction

Since late 2019, COVID-19 has transformed mRNA vaccines from a promising experimental modality into a clinically deployed platform. The pivotal phase 3 trials of BNT162b2 and mRNA-1273 provided the first large-scale clinical evidence that nucleoside-modified mRNA formulated in lipid nanoparticles (LNPs) could achieve high efficacy against symptomatic COVID-19 with an acceptable short-term safety profile (1, 2). Beyond these initial efficacy data, mRNA vaccines also demonstrated practical platform advantages: rapid sequence-to-product redesign, standardized cell-free manufacturing, avoidance of genomic integration, and comparatively straightforward antigen updating (3–8). The COVID-19 experience therefore changed not only pandemic response capacity but also the broader trajectory of RNA medicines.

At the same time, the pandemic revealed that success should not be defined solely by speed of authorization. Current COVID-19 mRNA vaccines showed strong and durable protection against severe disease, but they also exposed major biological and implementation challenges, including waning protection against infection, immune escape by rapidly evolving variants, limited induction of upper-airway mucosal immunity, and the recurrent need for booster immunization (9–15). These challenges, rather than diminishing the importance of the platform, have become the main drivers of second-generation vaccine design.

The goal of this review is therefore not simply to recatalogue the field, but to connect clinical performance with platform innovation. We first summarize the clinical performance of currently deployed COVID-19 mRNA vaccines, then compare mRNA with other COVID-19 vaccine platforms, highlight the key immunological and practical limitations revealed during variant evolution, and finally discuss the technological strategies being developed to address these limitations and extend mRNA applications beyond SARS-CoV-2.

## 2 Clinical performance of current COVID-19 mRNA vaccines

### 2.1 Effectiveness across SARS-CoV-2 variants

First-generation COVID-19 mRNA vaccines showed outstanding early performance against the ancestral virus and early variants. The pivotal BNT162b2 and mRNA-1273 trials established high efficacy against symptomatic COVID-19 in the pre-Omicron setting (1, 2), and subsequent observational studies during the Alpha and Delta periods showed high protection against symptomatic disease, hospitalization, and death, supporting mRNA vaccination as a central public-health intervention during the acute pandemic phase (12, 16–19).

That performance did not remain static. With the emergence of Omicron and its sublineages, vaccine effectiveness against infection and mild disease declined substantially because of both immune escape and waning antibody titers. However, protection against hospitalization and death remained substantially more durable, especially after booster doses and updated formulations targeting Omicron-descended lineages such as XBB.1.5 and JN.1 (9, 12–15, 20–24). This distinction between protection against infection and protection against severe outcomes should be stated explicitly, because treating these endpoints as interchangeable has contributed to confusion in parts of the literature.

Moreover, effectiveness estimates should be interpreted with caution because they are shaped by prior infection, hybrid immunity, testing behavior, age, risk profile, and study design. Observational estimates can therefore vary meaningfully across populations and time periods even when the biologic performance of the vaccine platform is broadly consistent (25–30). To avoid treating VE as a single fixed number, **Table 1** summarizes representative effectiveness estimates by variant period, vaccine product or regimen, clinical endpoint, and main interpretation.

**Table 1:** Representative effectiveness estimates of COVID-19 mRNA vaccination across variant periods.

Variant period	Product/regimen	Endpoint	Representative VE (95% CI)	Main interpretation
Alpha/early Delta	BNT162b2 or pooled mRNA, 2-dose primary series	Symptomatic disease/hospitalization	About 88% symptomatic; about 85% hospitalization (16, 17).	Very high early protection against matched or near-matched variants.
Delta	BNT162b2 or mRNA-1273, 2-dose primary series	Severe disease	BNT162b2: 93.4%; mRNA-1273: 96.1% (19).	Protection against severe outcomes remained strong despite early immune escape.
Omicron BA.1/BA.2	Pooled mRNA, >150 days after primary series	ED/UC/hospitalization	37% ED/UC; 54% hospitalization (179).	Marked loss of protection against infection after Omicron plus waning immunity.
Omicron BA.1/BA.2	Pooled mRNA after 3rd dose	ED/UC/hospitalization	66% ED/UC; 88% hospitalization (179).	Booster vaccination restored protection, especially against severe disease.
Omicron BA.4/BA.5	BNT162b2 BA.4/BA.5 bivalent booster	Hospitalization	58.7% (43.7-69.8%) (180).	Variant-updated boosters improved protection compared with waned original formulations.
XBB.1.5 period	2023–2024 XBB.1.5 vaccines (product-specific or pooled analyses)	Symptomatic disease/hospitalization	Around 41-55% symptomatic; around 54-65% hospitalization depending on study (13, 14, 21, 181).	Moderate but still useful short-term protection during antigenically drifted waves.
JN.1 period	JN.1-adapted BNT162b2 or mRNA-1273 boosters	Hospitalization/death	70.2% hospitalization; 76.2% death in Danish registry data (15).	Annual or periodic strain-updated boosting remains beneficial for high-risk groups.

VE values are representative rather than exhaustive and should be interpreted in light of age, prior infection, endpoint definition, and study design. Product attribution is specified where source studies were product-specific; otherwise pooled mRNA analyses are indicated explicitly.

### 2.2 Safety profile and public-health impact

Post-authorization surveillance supports a favorable overall safety profile for COVID-19 mRNA vaccines. Most adverse events are mild and self-limited, including injection-site pain, fatigue, headache, myalgia, and transient fever, and these reactions are consistent with short-lived innate immune activation after vaccination (31–34). Rare serious adverse events, particularly myocarditis and pericarditis, have been reported after mRNA vaccination and require risk-stratified communication and continued follow-up; population-level studies indicate that their overall prognosis is generally better than that of infection-associated cardiac inflammation (33–38).

From a public-health perspective, the benefits were substantial. Modeling studies and registry-based analyses indicate that COVID-19 vaccination prevented large numbers of deaths globally and remains most cost-effective in older adults and other high-risk groups, supporting a risk-stratified booster strategy rather than a one-size-fits-all approach for all age groups (39–42).

### 2.3 Clinical limitations informing next-generation vaccine design

The main limitations revealed by clinical use are now clear: protection against infection wanes faster than protection against severe disease; variant evolution can outpace the breadth of neutralizing antibodies elicited by prior formulations; repeated boosting is operationally difficult at population scale; and intramuscular administration does not reliably establish durable sterilizing immunity in the upper airway (9–13). These limitations set the agenda for the next phase of RNA-vaccine development and provide the rationale for the following sections, which examine the comparative platform advantages of mRNA during the pandemic and how RNA engineering, delivery innovation, immune shaping, and next-generation RNA formats may address the remaining gaps.

## 3 Comparative positioning of mRNA among COVID-19 vaccine platforms

Among the vaccine platforms deployed during the COVID-19 pandemic, mRNA did not become prominent because it was uniformly superior at every endpoint. Rather, it combined several advantages that were uniquely valuable during a rapidly evolving emergency: rapid antigen redesign, cell-free manufacturing, platform standardization across products, and relatively straightforward updating when new variants emerged (3, 7, 8).

Adenoviral-vector vaccines offered important benefits, including strong T-cell priming and easier refrigerated storage in some formulations, but they also faced challenges related to anti-vector immunity, lower flexibility for repeated homologous boosting, and slower strain updating (43, 44). Inactivated vaccines provided a familiar manufacturing pathway and broader whole-virion antigen exposure, but generally induced lower neutralizing-antibody titers and required repeated boosting (45). Protein-subunit vaccines offered attractive safety and storage profiles, yet usually depended on adjuvant optimization and longer antigen-production timelines (46). By contrast, mRNA was especially well suited to fast iterative redesign, which became decisive once antigenic drift accelerated (3, 7, 8, 12).

Thus, mRNA should be viewed not as a universally superior vaccine format, but as a rapid-response and readily updatable platform whose strengths are most evident when antigen redesign, standardized manufacturing, and iterative variant matching are required. The remaining challenge is to leverage these strengths while addressing observed weaknesses in durability, mucosal immunity, and deployment.

## 4 Immunological mechanisms and unresolved issues in repeated COVID-19 mRNA vaccination

### 4.1 Correlates of protection and persistence of severe-disease protection

**Figure 1:**
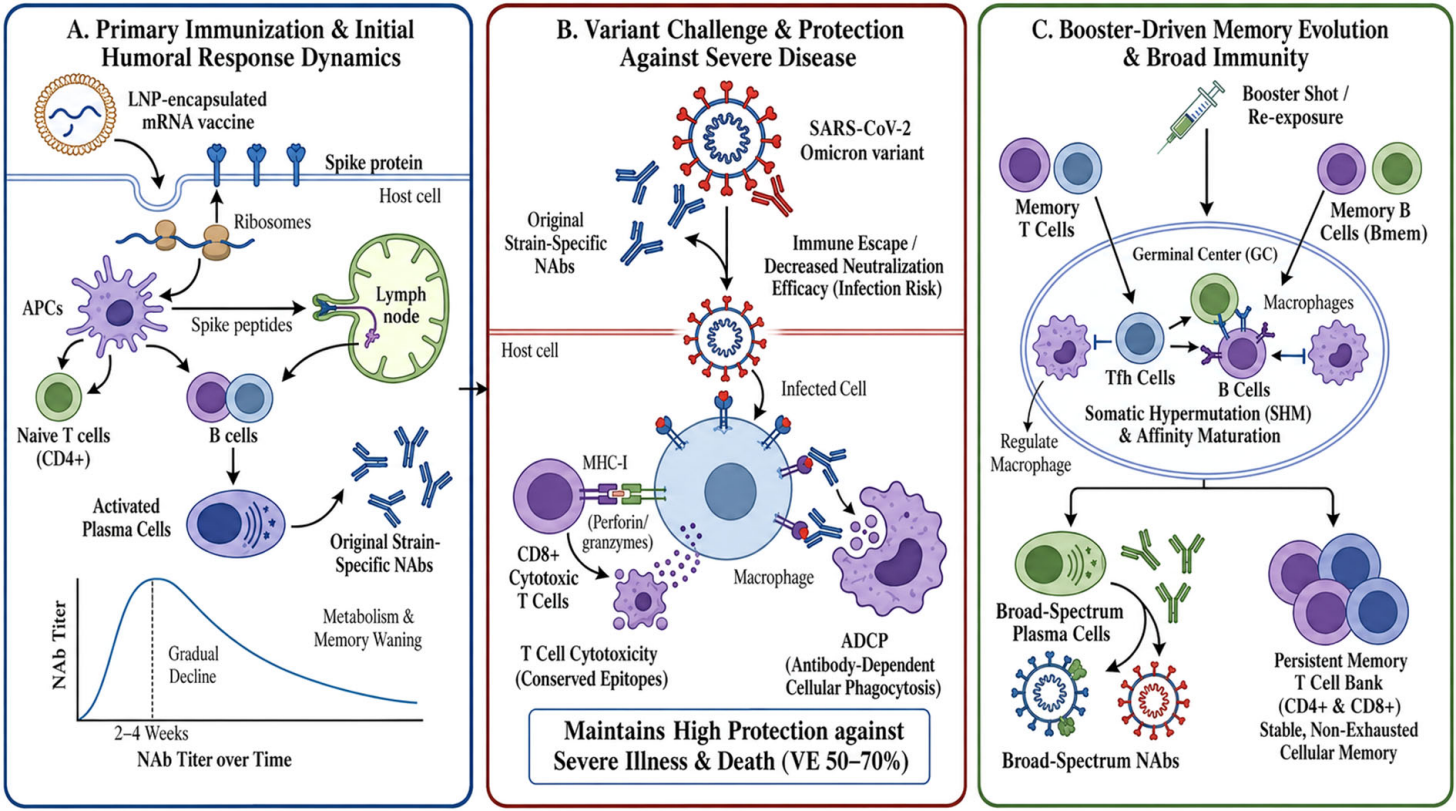
Immunological mechanisms of mRNA vaccine-induced protection. **(A)** Primary immunization with an LNP-encapsulated mRNA vaccine induces antigen expression, antigen presentation, and activation of naïve CD4+ T cells, B cells, and plasma cells, resulting in original strain-specific neutralizing antibodies that peak within 2–4 weeks and then gradually decline. **(B)** During SARS-CoV-2 variant challenge, reduced neutralization by original strain-specific antibodies can increase infection risk; however, conserved-epitope cytotoxic T cell responses, macrophage-mediated clearance, and antibody-dependent cellular phagocytosis help maintain protection against severe illness and death. **(C)** Booster vaccination or re-exposure promotes germinal-center activity, memory B- and T-cell evolution, somatic hypermutation and affinity maturation, broad-spectrum neutralizing antibodies, and persistent cellular memory, thereby supporting broader and more durable immunity.

Although no single immune marker fully predicts protection across all COVID-19 endpoints, neutralizing antibody levels remain the best-supported correlate or surrogate marker for short-term protection against infection and symptomatic disease at the population level (9, 47–51). The coordinated immune mechanisms underlying both early protection and persistent severe-disease protection are summarized in **Figure 1**. After primary immunization or boosting, antibody titers rise quickly and correlate with short-term protection against symptomatic infection, but they also decline over time and are strongly affected by spike mutations in antigenically distant variants (9, 47–50). This is why breakthrough infection became increasingly common after Omicron despite continued vaccination.

However, reduced protection against infection does not imply immunologic failure. Repeated vaccination and/or breakthrough infection continue to refine germinal-center and memory-B-cell responses, increasing clonal continuity, affinity maturation, and recognition of conserved epitopes across variants (52–55). When neutralization is incomplete after variant challenge, conserved-epitope T-cell responses and Fc-mediated non-neutralizing antibody functions can still contribute to infected-cell clearance and disease attenuation (56–60). Together, these mechanisms help explain why protection against hospitalization and death has remained more stable than protection against infection.

Thus, one of the main lessons from COVID-19 is that correlates of protection are endpoint-specific: neutralizing antibodies are most informative for infection risk, whereas memory B cells, T cells, and non-neutralizing effector mechanisms are central to sustained protection against severe disease. This framework is well supported, even though the quantitative thresholds needed for different endpoints remain under active study (9, 47, 59).

### 4.2 Immune imprinting

Immune imprinting has emerged as an important consideration for repeated SARS-CoV-2 vaccination, but its implications are more nuanced than a simple loss-of-function model. Historically, the related concept of original antigenic sin was introduced by Francis in 1960 to describe the tendency of prior antigenic exposure to shape responses to later variant antigens (61, 62). In the COVID-19 setting, prior infection or vaccination can bias recall toward previously seen epitopes, especially within conserved regions of spike, which may reduce the *de novo* recruitment of responses to newly emerged variant-specific epitopes (61, 63–65).

At the same time, imprinting is not uniformly detrimental. Cross-reactive memory can also serve as a useful scaffold for rapid recall, affinity maturation, and the generation of broader antibodies after boosting or breakthrough infection (66, 67). The key unresolved question is therefore not whether imprinting exists—it clearly does—but under what antigenic distances, dosing intervals, and exposure histories it becomes mainly constraining rather than beneficial. Variant-updated boosters can partly redirect the response, but they do not erase immunologic history (52, 65, 67).

### 4.3 IgG4 class switching

Repeated exposure to spike antigen through mRNA vaccination has been associated in some cohorts with an increase in spike-specific IgG4. This observation is reproducible at the serological level, especially after repeated antigen exposure, but its clinical meaning remains unsettled (68–70). It would therefore be premature to frame IgG4 switching as either clearly harmful or clearly beneficial.

On one hand, IgG4 has weaker canonical pro-inflammatory Fc functions than IgG1, including reduced Fcγ receptor and complement-mediated effector activation (71), which has led to concern that high IgG4 levels might reduce certain effector functions important for viral clearance. On the other hand, the consequences of subclass redistribution depend on the broader antibody repertoire, total antibody titer, epitope specificity, Fc-receptor context, and the infecting variant. Current evidence supports treating IgG4 as an informative immunologic signature of repeated exposure rather than as an established mechanism of reduced vaccine effectiveness (68–70).

### 4.4 Mucosal immunity

Intramuscular COVID-19 mRNA vaccines are highly effective at inducing systemic immunity but comparatively limited at generating durable upper-airway mucosal immunity. This mismatch helps explain why post-booster protection against severe disease can remain strong while infection and onward transmission are only incompletely prevented (10, 11, 72).

Intranasal and inhaled approaches are being explored to address this gap. Animal studies and early clinical data suggest that mucosal boosting can induce secretory IgA, lung- or airway-resident memory T cells, and more localized antiviral responses, especially in heterologous prime-boost settings (73–85). Nevertheless, this field is still evolving. It is not yet established that currently available mucosal strategies can reproducibly provide durable sterilizing immunity in humans, and formulation, delivery, and safety trade-offs remain major translational challenges (10, 75, 80–82). Overall, repeated COVID-19 mRNA vaccination has clarified that protection is endpoint-specific: neutralizing antibodies are central to short-term protection against infection, whereas memory B cells, T cells, and non-neutralizing effector mechanisms help sustain protection against severe disease.

## 5 Technological innovations addressing current limitations

### 5.1 RNA engineering and antigen design

**Figure 2:**
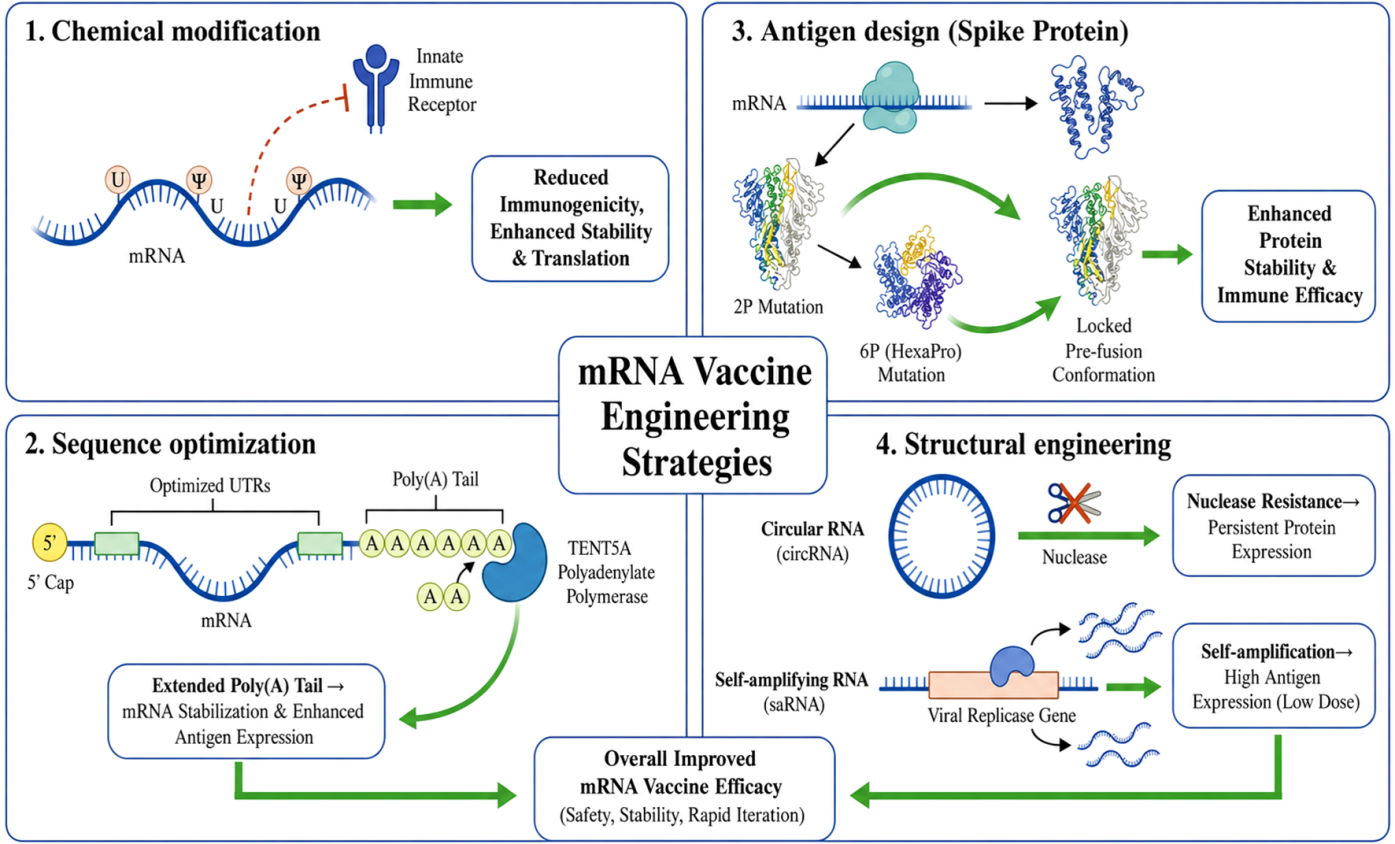
Schematic representation of mRNA vaccine engineering strategies.

The success of first-generation COVID-19 mRNA vaccines rests on precise engineering of both the RNA molecule and the encoded antigen, as illustrated in **Figure 2**. At the RNA level, nucleoside modification, cap optimization, untranslated-region design, and poly(A)-tail engineering improve translation efficiency, limit undesirable innate sensing, and increase stability. The foundational study by Karikó and colleagues showed that modified nucleosides such as pseudouridine suppress Toll-like receptor-mediated recognition of exogenous RNA, a finding that helped enable modern nucleoside-modified mRNA vaccines (86, 87). Subsequent work has further refined cap, UTR, and tail design, and has shown that host factors such as TENT5A can enhance vaccine performance by re-adenylating vaccine mRNA after delivery (4, 88).

At the antigen-design level, structural biology rapidly translated into vaccine engineering. The introduction of the prefusion-stabilizing 2P mutation locked spike in an immunogenically favorable conformation, and the later HexaPro/6P design added further proline substitutions to improve stability and expression, as shown schematically in **Figure 2** (89, 90). Newer work suggests that in some contexts non-stabilized spike antigens may broaden epitope targeting and promote antibodies to conserved regions, emphasizing that maximally stable antigen design is not always synonymous with maximally broad immunity (91).

### 5.2 Delivery-system innovation

**Figure 3:**
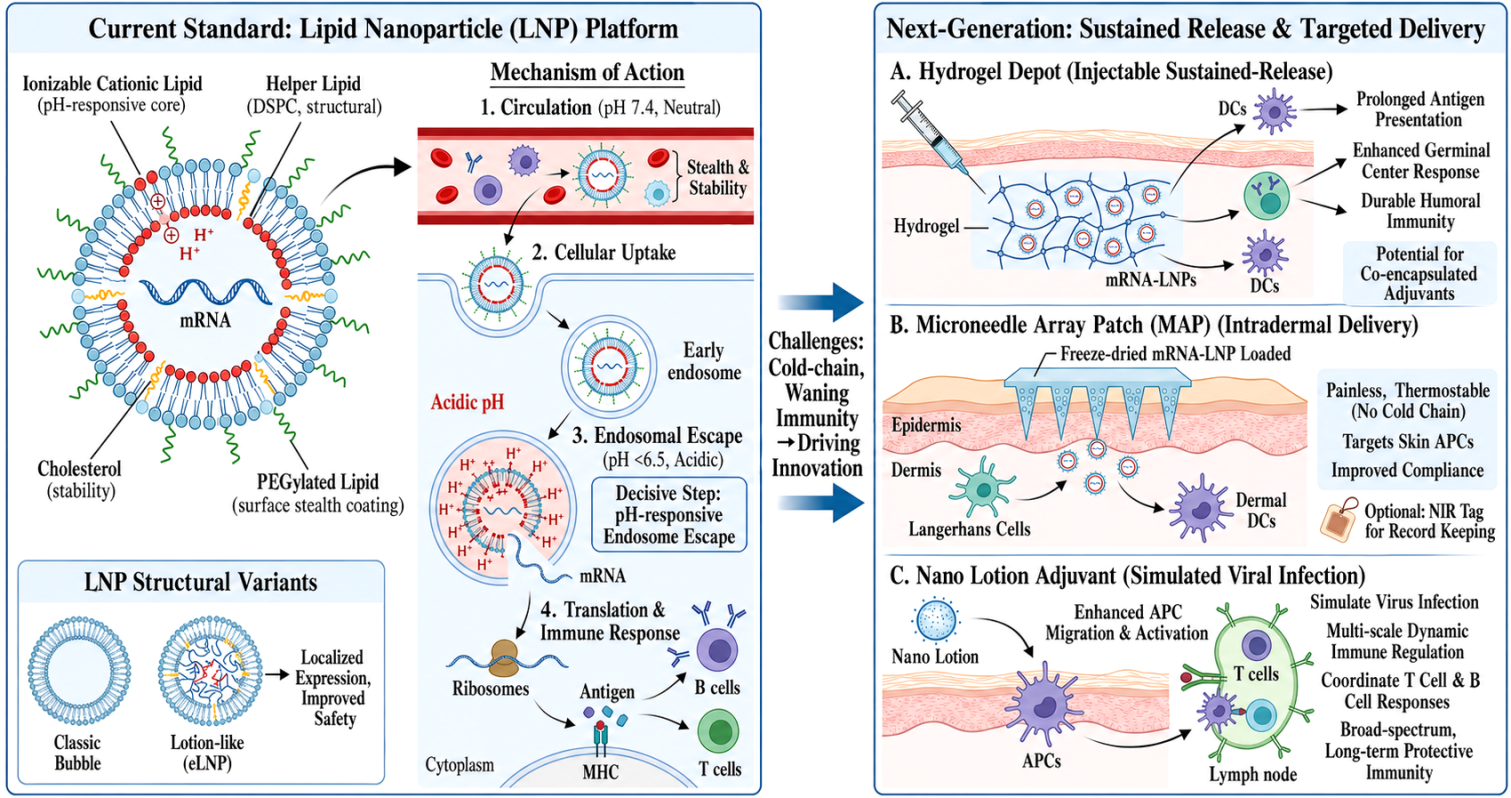
Delivery platforms for mRNA vaccines: lipid nanoparticles (LNP) mechanisms and next-generation sustained-release strategies.

LNPs remain the clinically dominant delivery system for mRNA vaccines because they solve the two central delivery problems of RNA therapeutics: nuclease instability and inefficient cytosolic entry. Their four-component architecture—ionizable lipid, phospholipid, cholesterol, and PEG-lipid—supports RNA encapsulation, tissue delivery, and endosomal escape (92–100). **Figure 3** depicts this delivery process from LNP composition and cellular uptake to acidic endosomal escape, cytosolic mRNA release, antigen translation, and immune activation. Ionizable lipids exhibit pH-responsive behavior: they remain largely neutral at physiological pH but become protonated in acidic endosomes, thereby promoting endosomal membrane destabilization and cytosolic release of mRNA (95–98).

Current work is focused on improving the balance between potency, biodistribution, tolerability, and manufacturability. Representative strategies include biodegradable ionizable lipids, AI-guided lipid design, PEG alternatives, and LNP structural tuning to favor more localized antigen expression (96–101). At the same time, next-generation delivery formats such as hydrogels, microneedle patches, and nanoemulsions are being explored to extend antigen exposure, reduce cold-chain dependence, simplify administration, or better target antigen-presenting tissues (102–109). These approaches are not intended simply as replacements for LNPs; rather, as summarized in **Table 2** and illustrated in the right panel of **Figure 3**, they address specific delivery bottlenecks such as sustained antigen exposure, skin/APC targeting, administration convenience, and mucosal or immune-programming potential.

**Table 2:** Representative delivery strategies for RNA vaccines.

Delivery system	Key advantages	Main translational challenges	Representative notes/examples
Lipid nanoparticles (LNPs)	Highest clinical maturity; efficient encapsulation, uptake, and endosomal escape; adaptable lipid chemistry.	Reactogenicity, biodistribution control, PEG-related concerns, and continued optimization of potency versus tolerability.	Core platform for approved COVID-19 mRNA vaccines; intense work on ionizable lipids and PEG alternatives (92–101).
Hydrogel depots	Sustained antigen release, prolonged germinal-center stimulation, and opportunities for local immune programming.	Limited clinical data and more complex manufacturing for specialized depot architectures.	Attractive for dose sparing or single-dose strategies and for therapeutic vaccines (102–105).
Microneedle patches	Needle-sparing, skin-targeted delivery, improved convenience, and potential thermostability benefits with dried formulations.	Patch fabrication, formulation stability, and consistent skin delivery across users require further optimization.	May improve access, facilitate simplified administration records, and support skin/APC-targeted vaccination approaches (106, 107, 182).
Nanoemulsions/mucosal carriers	Can improve immune programming, APC recruitment, and possibly mucosal targeting in future respiratory vaccines.	Formulation stability, reproducibility, and translational evidence remain less mature than for LNPs.	Most useful as emerging adjunctive or specialized delivery formats rather than replacements for LNPs (80–82, 108, 109).

### 5.3 Adjuvants and immune-shaping strategies

Although mRNA-LNP formulations already possess intrinsic adjuvant activity, next-generation design is moving from generic self-adjuvancy toward more deliberate immune shaping. Co-delivery of cytokine-encoding RNAs such as IL-12 or IL-7 can enhance cytotoxic T-cell responses or support longer-lasting humoral responses, while integration of innate agonists such as STING-activating materials may improve lymphatic delivery and T-cell priming (110–112). The conceptual shift is important: the aim is no longer only to generate a strong response, but to generate the right response for the desired clinical endpoint.

Clinical translation of this concept is already visible in local tumor immunotherapy. Intratumoral MEDI1191, an mRNA encoding IL-12, has been evaluated with durvalumab in patients with advanced solid tumors (113), and mRNA-2752 delivers mRNAs encoding OX40L, IL-23, and IL-36γ for intratumoral administration either alone or in combination with durvalumab (114). More recently, STX-001 extended this approach to an LNP-encapsulated self-replicating mRNA encoding IL-12 for intratumoral administration (115). These examples illustrate that immune-shaping RNA is not limited to vaccine antigen delivery, but can also be used to remodel the tumor microenvironment through transient local cytokine expression.

### 5.4 Next-generation RNA platforms and broader variant coverage

**Figure 4:**
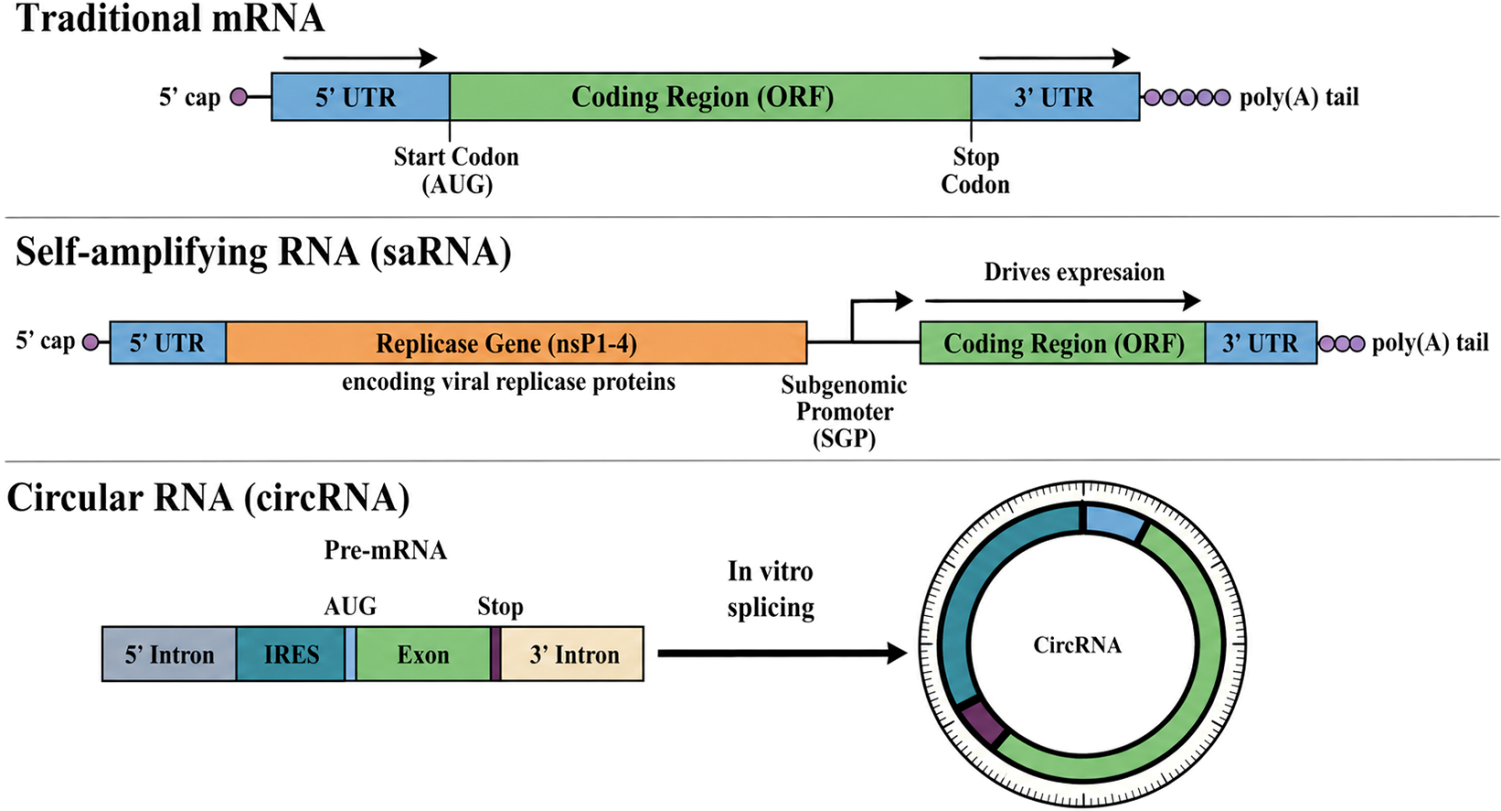
Schematic of traditional mRNA, saRNA, and circRNA structures.

Next-generation RNA platforms should be discussed according to developmental maturity. **Figure 4** and **Table 3** together summarize the structural and translational differences among conventional mRNA, saRNA, and circRNA platforms. Self-amplifying RNA (saRNA) is currently the more clinically advanced modality. By encoding both the target immunogen and a viral replicase complex, saRNA amplifies intracellular RNA copies and can achieve strong immunogenicity at substantially lower doses than conventional mRNA. Clinical studies of ARCT-154 showed favorable safety, immunogenicity, and efficacy, and follow-up analyses reported durable immune responses up to 12 months in comparison with BNT162b2 (116–118). Importantly, ARCT-154/Kostaive has already received regulatory approval in Japan and the European Union, making saRNA a clinically realized platform rather than an early-stage concept (119, 120).

**Table 3:** Comparison of RNA platforms relevant to COVID-19 vaccine development.

Platform	Main strengths	Main limitations	Current maturity/examples
Conventional mRNA	Clinically validated; rapid redesign; scalable cell-free manufacturing; strong immunogenicity with nucleoside-modified RNA/LNP formulations.	Higher dose than saRNA in many settings; cold-chain dependence; protection against infection wanes; limited mucosal immunity after intramuscular delivery.	Established products include BNT162b2 and mRNA-1273 (1, 2).
Circular RNA (circRNA)	Closed-loop architecture improves nuclease resistance and may support prolonged protein expression.	Translation efficiency, purification, and large-scale manufacturing remain under optimization; limited clinical vaccine evidence.	Promising but still largely preclinical/early translational for COVID-19 vaccines (4, 128).
Self-amplifying RNA (saRNA)	Intracellular amplification enables low-dose vaccination, efficient antigen expression, and potential durability advantages.	Larger constructs and product-specific manufacturing/control challenges; not all backbones behave equivalently and formulation still matters.	Clinically advanced: ARCT-154/Kostaive approved in Japan/EU; GEMCOVAC-OM authorized in India; HDT-301 is clinical-stage (116–124).

Additional examples reinforce this point. GEMCOVAC-OM, an Omicron-specific thermostable self-amplifying mRNA booster delivered intradermally, received emergency authorization in India and showed favorable phase 2/3 immunogenicity and safety data (121). Other programs, such as HDT-301, illustrate that platform diversity extends beyond a single product lineage and includes clinical-stage repRNA candidates with distinct formulation and backbone designs (122). Clinical phase 1 studies of optimized self-replicating RNA platforms have also shown that very low-dose or single-dose vaccination can be immunogenic and that durability can be comparable to or better than a licensed comparator vaccine in another infectious-disease setting (123, 124). The diversity of saRNA backbones is also expanding. Comparative and mechanistic studies indicate that alphaviral backbone choice can shape expression, innate sensing, and immunogenicity, supporting a move away from a one-backbone-fits-all view of the platform (125, 126). This point is reinforced by a recent EEEV-derived self-replicating RNA platform developed for combination cancer immunotherapies, which demonstrated the importance of empirically selecting fit-for-purpose srRNA vectors for different therapeutic applications (127).

By contrast, circular RNA (circRNA) remains largely preclinical in the vaccine field. Its closed-loop structure is attractive because it resists exonuclease degradation and may support longer protein expression, but clinical evidence for COVID-19 circRNA vaccines remains limited compared with saRNA (128). Thus, the key distinction is not only architectural but also translational: as reflected in **Figure 4** and **Table 3**, circRNA should be described as a promising emergent architecture, not as a platform currently matched to saRNA in clinical maturity.

Beyond changes in RNA format, next-generation COVID-19 vaccine design also includes multivalent and pan-sarbecovirus strategies. These include updated bivalent or monovalent variant-matched vaccines, mosaic nanoparticle displays, and immunogens targeting conserved regions such as the S2 subunit and fusion machinery (129–134). Such approaches seek to reduce the need for frequent strain chasing and to provide broader resilience against future coronavirus evolution. Collectively, next-generation RNA-vaccine development is shifting from maximizing antigen expression alone toward optimizing the match among RNA architecture, antigen breadth, delivery site, innate immune tuning, and clinical objective.

## 6 Vaccination strategies for special populations

Vaccination strategies for special populations should be guided by baseline risk, expected immune responsiveness, exposure risk, and the safety profile of available formulations rather than by a uniform age-independent schedule. **Table 4** summarizes how this principle applies to immunocompromised individuals, pregnant and lactating women, older adults, and children/adolescents, while the subsections below explain the evidence behind these practical implications.

**Table 4:** Practical considerations for COVID-19 mRNA vaccination in special populations.

Population	Main issue	Practical vaccination implication	Supporting evidence
Immunocompromised individuals	Blunted or heterogeneous humoral responses, especially after B-cell-depleting therapy or transplantation.	Use updated boosters, consider additional doses in selected groups, and individualize follow-up or serologic assessment when clinically justified.	(135–144)
Pregnant and lactating women	Need to balance maternal protection with fetal/infant safety concerns.	Current evidence supports vaccination; maternal antibodies can be transferred transplacentally and through breast milk.	Safety/birth outcomes (145, 146); trial-inclusion modeling (147):; early-childhood, lactation, antibody-transfer, and guidance evidence (148–152):.
Older adults	Immunosenescence reduces response magnitude and durability.	This group derives the clearest benefit from risk-based booster strategies with updated formulations.	(14, 15, 153–156)
Children and adolescents	Lower baseline risk of severe acute disease but non-zero risk of complications and post-COVID conditions.	Age- and risk-sensitive vaccination remains reasonable, with benefits extending beyond acute infection prevention in some studies.	(157–161)

### 6.1 Immunocompromised populations

Immunocompromised populations remain among the clearest examples of why vaccination policy must be individualized. Solid-organ transplant recipients, patients receiving B-cell-depleting therapies, and some individuals with hematologic malignancies often show markedly blunted humoral responses to COVID-19 mRNA vaccines, although T-cell responses may be partially retained in some subgroups (135–141). Updated boosters and additional doses can improve responses in part of this population, but heterogeneity remains large, making serologic monitoring and risk-stratified scheduling reasonable in selected high-risk settings (136, 141–144).

### 6.2 Pregnancy and lactation

Evidence to date supports COVID-19 mRNA vaccination during pregnancy and lactation. Large cohort and meta-analytic data have not shown increases in major adverse birth outcomes attributable to vaccination (145, 146). Modeling work further highlights the potential health costs of excluding pregnant participants from vaccine trials, supporting more deliberate inclusion where ethically appropriate (147). Additional population-level, immunologic, and guidance evidence indicates that vaccination during pregnancy and lactation is compatible with favorable early-childhood outcomes and can transfer protective antibodies through the placenta and breast milk (148–152).

### 6.3 Older adults

In older adults, immunosenescence reduces the magnitude and speed of vaccine responses, but booster vaccination remains clinically valuable. Updated boosters improve neutralizing titers and reduce hospitalization and death, making older age groups the strongest candidates for continued risk-based seasonal or periodic boosting (14, 15, 153–156).

### 6.4 Children and adolescents

In children and adolescents, the risk-benefit profile differs from that in older adults but still favors vaccination in appropriate settings. Available evidence supports good safety and benefits that extend beyond prevention of acute disease to reduction in post-COVID condition risk in some studies, although policy decisions should remain age- and risk-sensitive (157–161).

## 7 Expansion of mRNA technology beyond COVID-19

Beyond COVID-19, RSV provides one of the clearest examples that mRNA vaccines have become a broader clinical platform. Moderna’s mRESVIA (mRNA-1345), a nucleoside-modified mRNA vaccine encoding the prefusion-stabilized RSV F glycoprotein, was initially approved by the FDA in 2024 for the prevention of RSV-associated lower respiratory tract disease in adults aged 60 years and older, with the current label also including adults aged 18–59 years who are at increased risk for RSV-associated lower respiratory tract disease (162, 163). This milestone demonstrates that mRNA vaccine development has moved beyond COVID-19 indications and can be translated to other respiratory pathogens.

In oncology, mRNA technology is being developed for individualized neoantigen vaccines, shared tumor-antigen vaccines, and combinations with immune checkpoint blockade (164–170). Several individualized mRNA vaccine programs have now provided clinical proof-of-concept, including mRNA-4157/V940 in combination with pembrolizumab in resected high-risk melanoma and autogene cevumeran/BNT122 in pancreatic ductal adenocarcinoma, both of which support the feasibility of patient-specific vaccine manufacture and the induction of tumor-reactive T-cell responses (165–167). In parallel, self-amplifying RNA platforms are also being explored. A notable example is the Gritstone bio program, in which chimpanzee adenoviral priming was combined with self-amplifying mRNA neoantigen boosting in patients with advanced solid tumors; phase 1 interim data from this ongoing phase 1/2 study showed feasible manufacturing, acceptable safety, and durable neoantigen-specific CD8 T-cell responses (171). Despite these advances, individualized cancer vaccines remain clinically challenging because efficacy depends on neoantigen (neoAg) selection, tumor heterogeneity, the pre-existing “hot” versus “cold” tumor microenvironment, manufacturing turnaround time, and rational combination with checkpoint inhibitors.

The same platform logic is also being extended to other infectious diseases, including influenza, HIV, malaria, and Zika (172–177). For influenza, the main attraction of mRNA is rapid antigen updating, which may be useful when vaccine strains need to be matched to evolving seasonal or zoonotic viruses. For HIV, mRNA provides a flexible way to express complex envelope immunogens, including virus-like particle-forming designs, although safety, reactogenicity, and the difficulty of eliciting broadly neutralizing antibodies remain major barriers. For malaria and Zika, mRNA-based strategies remain less clinically mature than COVID-19 or RSV vaccines, but they illustrate how the platform can be adapted to pathogens requiring different antigen formats and immune profiles.

The application of mRNA technology also extends beyond preventive vaccination. In oncology, locally delivered mRNA can be used as an immunomodulatory drug by transiently expressing cytokines or immune-stimulatory ligands within the tumor microenvironment. Intratumoral MEDI1191, which encodes IL-12, has been evaluated with durvalumab in patients with advanced solid tumors (113), and mRNA-2752 delivers OX40L, IL-23, and IL-36γ mRNAs alone or in combination with durvalumab (114). These clinical examples show that mRNA can be used not only to encode antigens, but also to reshape the tumor microenvironment through localized cytokine or immune-stimulatory protein expression.

Self-replicating RNA is also entering this therapeutic space. STX-001 is an LNP-encapsulated self-replicating mRNA encoding IL-12 for intratumoral administration in advanced solid tumors (115), and recent EEEV-derived srRNA platforms have been developed for combination cancer immunotherapies (127). These approaches illustrate a broader therapeutic logic: saRNA may be useful when sustained local expression of an immunomodulatory payload is desired, but clinical translation still requires careful control of tissue localization, dose, durability, inflammatory toxicity, and compatibility with checkpoint inhibitors.

Beyond oncology, therapeutic protein-expression approaches aim to deliver mRNA *in vivo* so that host cells transiently produce functional proteins, antibodies, or other therapeutic molecules (5, 93, 178). Across these indications, the key translational questions remain similar: delivery to the right tissue, adequate durability, acceptable reactogenicity, scalable manufacturing, and alignment between the immunologic or protein-expression profile of the platform and the clinical problem being addressed. Thus, the broader value of mRNA technology lies not in a single disease area, but in its modularity: the same core platform can be redirected toward vaccines, cancer immunotherapy, cytokine-based immune modulation, and selected protein-replacement applications, provided that delivery and safety requirements are matched to the intended use.

## 8 Conclusion and prospect

The COVID-19 experience established mRNA vaccines as a practical clinical platform rather than a purely experimental idea. The pivotal BNT162b2 and mRNA-1273 trials and subsequent observational studies showed that first-generation nucleoside-modified mRNA/LNP vaccines can provide strong protection against symptomatic disease and especially severe outcomes (1, 2, 12, 16–24). At the same time, waning protection against infection and antigenic drift (9–15), incomplete upper-airway mucosal immunity (72–85), and heterogeneous responses across immunocompromised, pregnant or lactating, older, and pediatric populations (135–161) have redefined the research agenda.

These limitations have shifted the field from first-generation clinical success toward second-generation optimization. Current strategies include improved RNA chemistry and antigen design (86–91), more targeted delivery systems and deliberate immune-shaping approaches (92–112), and platform diversification into clinically advanced saRNA and still-emerging circRNA approaches (116–128). Protection against infection, protection against transmission, and protection against severe disease should therefore not be treated as interchangeable goals, because each endpoint may require a different balance of antigen breadth, antibody durability, tissue localization, T-cell engagement, and booster timing.

The immunological questions raised by repeated COVID-19 mRNA vaccination also remain central. Neutralizing antibodies are still the most informative correlate for short-term protection against infection (47–50), whereas memory B cells, T cells, and non-neutralizing antibody functions help explain the persistence of protection against severe disease (52–60). At the same time, immune imprinting, IgG4 class switching, and limited mucosal immunity should be viewed as active areas of investigation rather than settled explanations for vaccine performance (61–70, 72–85). A more precise understanding of these mechanisms will be essential for designing vaccines that are not only immunogenic, but also durable, broadly protective, and appropriate for different populations.

Next-generation RNA platforms will also require careful distinction between technological promise and translational maturity. Conventional modified mRNA remains the most clinically established format, while saRNA has now crossed an important translational threshold through clinically advanced and authorized COVID-19 vaccine products (116–124). By contrast, circRNA remains a promising but earlier-stage approach whose potential advantages in stability and expression duration still require clinical validation (128). Future platform development should therefore avoid treating all RNA formats as equally mature and should instead define which architecture is best suited to each clinical objective.

More broadly, COVID-19 provided a template for how RNA medicines can be iterated under real clinical pressure. The emergence of mRESVIA (162, 163), the expansion of individualized cancer-vaccine programs including conventional mRNA and saRNA approaches (164–171), cytokine-based tumor immunotherapy with mRNA and self-replicating RNA payloads (113–115, 127), continued adaptation of mRNA platforms to influenza, HIV, malaria, Zika, and therapeutic protein-expression applications (172–178), and the clinical exploration of special-population strategies (135–161) all suggest that the legacy of COVID-19 mRNA vaccines will extend well beyond SARS-CoV-2. The central challenge ahead is therefore no longer whether RNA platforms can work, but how to match the right RNA architecture, antigen or therapeutic payload, delivery system, immune-shaping strategy, dosing schedule, and target population to the biological problem at hand. Meeting this challenge will determine whether mRNA technology becomes not only a rapid-response vaccine platform, but a broadly adaptable class of RNA medicines.
